# High-level production of animal-free recombinant transferrin from *saccharomyces cerevisiae*

**DOI:** 10.1186/1475-2859-9-87

**Published:** 2010-11-17

**Authors:** Christopher JA Finnis, Tom Payne, Joanna Hay, Neil Dodsworth, Diane Wilkinson, Philip Morton, Malcolm J Saxton, David J Tooth, Robert W Evans, Hans Goldenberg, Barbara Scheiber-Mojdehkar, Nina Ternes, Darrell Sleep

**Affiliations:** 1 Novozymes Biopharma UK Limited, Castle Court, 59 Castle Boulevard, Nottingham, NG7 1FD, UK; 2 School of Biomedical Sciences, Faculty of Medicine & Health Sciences, University of Nottingham Medical School, Queen's Medical Centre, NG7 2UH, UK; 3 Metalloprotein Research Group, Division of Biosciences, School of Health Sciences and Social Care, Heinz Wolff Building, Brunel University, Uxbridge, Middlesex, UB8 3PH, UK; 4 Department of Medical Chemistry, Medical University of Vienna, Währingerstrasse 10, A-1090, Vienna, Austria

## Abstract

**Background:**

Animal-free recombinant proteins provide a safe and effective alternative to tissue or serum-derived products for both therapeutic and biomanufacturing applications. While recombinant insulin and albumin already exist to replace their human counterparts in cell culture media, until recently there has been no equivalent for serum transferrin.

**Results:**

The first microbial system for the high-level secretion of a recombinant transferrin (rTf) has been developed from *Saccharomyces cerevisiae *strains originally engineered for the commercial production of recombinant human albumin (Novozymes' Recombumin^® ^USP-NF) and albumin fusion proteins (Novozymes' albufuse^®^). A full-length non-N-linked glycosylated rTf was secreted at levels around ten-fold higher than from commonly used laboratory strains. Modification of the yeast 2 μm-based expression vector to allow overexpression of the ER chaperone, protein disulphide isomerase, further increased the secretion of rTf approximately twelve-fold in high cell density fermentation. The rTf produced was functionally equivalent to plasma-derived transferrin.

**Conclusions:**

A *Saccharomyces cerevisiae *expression system has enabled the cGMP manufacture of an animal-free rTf for industrial cell culture application without the risk of prion and viral contamination, and provides a high-quality platform for the development of transferrin-based therapeutics.

## Background

Transferrin (Tf) is the major iron binding protein in human plasma, responsible for the regulated delivery of iron to cells. It is a monomeric glycoprotein (~80 kDa) with the capacity to bind two ferric ions very tightly, but reversibly. Transferrin consists of two globular lobes (the N-lobe and C-lobe) each made up of two sub-domains separated by a deep cleft, which contains the binding site for a ferric ion and a synergistic carbonate anion. In the vast majority of cell types iron is acquired by the binding of iron-laden holo-transferrin to a specific transferrin receptor (TfR), followed by endocytosis of the Fe^3+^/Tf/TfR complex. Iron is released in the acidic conditions of the endosome, after which the Tf/TfR complex is returned to the cell surface, from where the iron-free apo-transferrin is released back into circulation [1].

A current major use for plasma-derived transferrin is in the preparation of media for the culture of mammalian cells. The use of such media for the production of pharmaceutical products requires stringent attention to the source materials to control the risk of viral and prion disease transmission. A recombinant microbial source avoiding the use of mammalian-derived materials provides an obvious advantage.

Therapeutic applications have also been proposed for transferrin [2,3]. Treatment of the rare disease hereditary atransferrinemia is the most obvious, where the beneficial effects of transferrin infusion have been described [4]. Recently, exogenous transferrin has been used to ameliorate disease in β-thalassemic mice, where improved erythropoiesis and red blood cell survival were observed after treatment with transferrin [5]. This suggests that transferrin therapy might provide a viable alternative to blood transfusion and chelation therapy for treating human β-thalassemia and other diseases with concurrent anemia and iron overload. Furthermore, transferrin has been proposed as a means of reducing free-iron concentrations and consequent oxidative damage and infection risk [6-8]. Since transferrin is relatively resistant to proteolytic degradation and the TfR is abundant on gastrointestinal epithelial cells, transferrin fusions and conjugates with GCSF, insulin and human growth hormone have been studied for oral delivery of therapeutic proteins [9,10]. The 7-10 day plasma half-life of fully glycosylated transferrin in humans and the 14-15 day half-life reported for carbohydrate-deficient transferrin also promise improved plasma half-life over the unfused proteins *in vivo *[11,12]. Finally, overexpression of the TfR by many cancer cells has promoted interest in the use of transferrin for tumor imaging and targeted drug delivery [1].

Glycosylated and non-glycosylated recombinant human transferrins have been secreted from baby hamster kidney (BHK) cells. Both were shown to be fully equivalent to the serum protein in iron binding and interaction with cellular transferrin receptors, with glycosylation being unimportant for transferrin function *in vitro *[13]. However, expression from mammalian cells does not meet the requirement for an inexpensive source of recombinant transferrin, and may itself rely upon an animal-derived source of transferrin in the culture media. Expression of full-length transferrin from microbial sources has previously only been reported at low levels ranging from 3-40 mg/L [14-16], although transferrin production by recombinant plants has recently been described at higher levels [17,18]. While this could satisfy the need for an animal-free transferrin source, MALDI MS analysis of the rice-derived transferrin showed two peaks, with the mass of the predominant peak appearing 1,392 Da higher than the predicted mass, suggesting post-translational modification.

Here we describe a highly productive yeast expression system using baker's yeast, *Saccharomyces cerevisiae*. Successive rounds of chemical mutagenesis and selection, combined with deletion of specific endogenous genes, have produced yeast strains with improved product yield and quality [19-22]. These host strains were combined with an episomal expression vector based upon the highly stable yeast 2 μm plasmid [23]. The expression vector contains the *LEU2 *selectable marker and a recombinant protein expression cassette, comprising the *PRB1 *promoter, a secretory leader, the coding sequence of interest and the *ADH1 *terminator. *S. cerevisiae *transformed with such vectors contains only yeast DNA and the coding sequence of interest. The yeast expression system was initially developed for the high-level secretion of recombinant human serum albumin (rHA), but studies have shown that this system can equally be used to express a diverse range of heterologous proteins. We have used this system as the basis for the development of a recombinant transferrin production platform.

## Results and discussion

### Removal of N-linked glycosylation sites in rTf reduces product heterogeneity and allows visualization by SDS-PAGE

**Figure 1:**
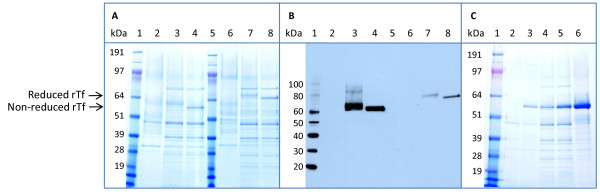
**Shake flask analysis of rTf expression. (A) **SDS-PAGE analysis for rTf with and without N-linked glycosylation. 30 μl of five-day BMMD SFC supernatant was analyzed per lane by SDS-PAGE (4-12% Bis-Tris NuPAGE^®^, MOPS buffer, Invitrogen). Samples in lanes 2-4 were non-reduced; the corresponding samples in lanes 6-8 were reduced. Track loadings were: Lanes 1 & 5) 5 μl SeeBlue^® ^Plus2 protein standards (Invitrogen) used for comparing relative migration of Tf samples only. Lanes 2 & 6) DYB7 [pSAC35] supernatant, negative control. Lanes 3 & 7) DYB7 [pDB2506] supernatant, glycosylated transferrin. Lanes 4 & 8) DYB7 [pDB2536] supernatant, rTf (N413Q, N611Q). **(B) **Western blot analysis for rTf with and without N-linked glycosylation. Lane 1) 5 μl MagicMark™ XP western protein standards (Invitrogen). All other lanes as for (A) above. **(C) **Relative productivity of rTf (N413Q, N611Q) expression systems showing the effect of *PDI1 *copy number enhancement. 30 μl of five-day BMMD SFC supernatant was analyzed per lane by non-reducing SDS-PAGE (4-12% Bis-Tris NuPAGE^®^, MOPS buffer, Invitrogen). Track loadings were: Lane 1) SeeBlue^® ^Plus2 protein standards (Invitrogen) used for comparing relative migration of Tf samples only. Lane 2) JRY188 [pDB2536] with one genomic *PDI1 *gene. Lane 3) JRY188 [pDB2711] with multiple copies of *PDI1*. Lane 4) DYB7 [pDB2536] with one genomic *PDI1 *gene. Lane 5) DP9 [pDB2536] with two genomic *PDI1 *genes. Lane 6) DYB7 [pDB2711] with multiple copies of *PDI1*.

Our initial transferrin expression vector (pDB2506) contained the mature human transferrin (C1 variant) sequence derived from a cDNA. Otherwise, the expression cassette (promoter, mHSA/*MFα1*-leader and terminator) was the same as for rHA production [19]. Analysis of shake flask culture (SFC) supernatants of DYB7 [pDB2506] by SDS-PAGE revealed a faint diffuse protein band (Figure 1, Lanes A3 and B3), indicating that product was secreted but that it was heterogeneous. Yeast hyper-mannosylation at the two N-linked glycosylation sites within the wild-type coding region was expected to result in a mixture of glycoforms and difficulty in visualization. Mutations were introduced to eliminate both N-linked glycosylation sites, substituting asparagine with glutamine at positions 413 and 611 of the mature protein. The molecular weight of non-glycosylated transferrin is approximately 75 kDa. After expression of rTf (N413Q, N611Q) from DYB7 [pDB2536], SDS-PAGE analysis showed a clearly visible band for non-glycosylated transferrin (Figure 1, Lanes A4 and B4). The identity of this band and the absence of proteolytic fragments were confirmed by western blotting (Figure 1B).

### *PDI1 *overexpression increases rTf production approximately ten-fold

**Figure 2:**
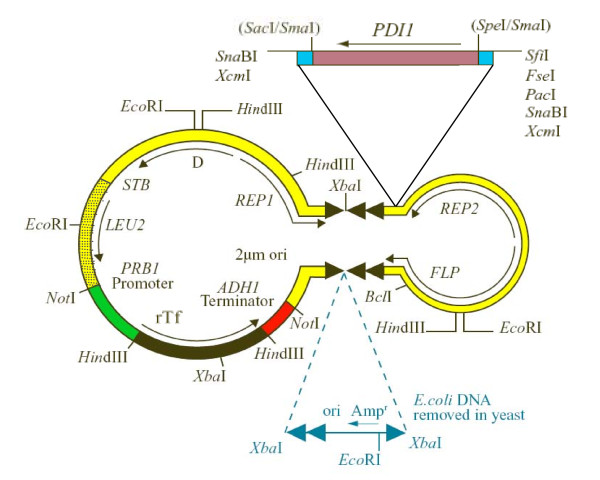
**A 2 μm-based plasmid for expression of rTf in yeast, illustrating the rTf expression cassette, the *PDI1 *gene and inserted *E. coli *DNA**. Native 2 μm sequences (yellow) are shown with gene transcripts (arrows). The *S. cerevisiae LEU2 *selectable marker (yellow shaded) and transferrin gene (green/black/red) are integrated between the *STB *locus and the origin of replication. Bacterial sequences flanked by two repeat sequences containing the recognition target for *FLP*-recombinase (blue) are integrated into an inverted repeat (black triangles) causing them to be eliminated in yeast. The *PDI1 *gene is inserted downstream of the *REP2 *gene.

High cell density fed-batch fermentation of DYB7 [pDB2536] produced a rTf yield of 0.17 g/L (Table 1). This demonstrated the feasibility of rTf (N413Q, N611Q) production in *S. cerevisiae*, but yields were low when compared to a similar system expressing rHA. Due to the known beneficial effects of *PDI1 *on protein production in yeast [24,25] a new strain, DP9, was constructed with an additional copy of *PDI1 *integrated at the *PDI1*-locus. This strain showed a significant increase in rTf production (Figure 1, Lane C5). In view of the high copy number and stability of whole 2 μm-derived plasmids, insertion of *PDI1 *into the plasmid was considered for increasing the *PDI1 *gene copy number. While it has previously been reported that insertion of *PDI1 *in the 2 μm plasmid was deleterious [24], we reasoned that the problem might be related to both the architecture of the 2 μm plasmid, consisting of several overlapping transcription units, and the location of the *PDI1 *insertion site (Figure 2). Most potential insertion sites are within functional regions concerned with plasmid amplification or partitioning. As a result, insertion of "foreign" DNA is likely to disrupt one of these important functions, thereby destabilizing the plasmid.

**Table 1:** rTf production titers in high cell density fed-batch fermentations

Strain	Number of fermentations	**Growth rate (h**^**-1**^**)**	pH	Transferrin titer (g/L in supernatant)*
DYB7 [pDB2536]	1	0.07	6.5	0.17

DP9 [pDB2536]	2	0.07	6.5	0.39, 0.31

DYB7 [pDB2711]	2	0.07	6.5	1.48, 1.52

DYB7 [pDB3235]	2	0.06	6.2	0.24, 0.13

DYB7 [pDB3237]	2	0.06	6.2	2.25, 2.33

* Supernatant concentrations determined by HPLC.

Novel sites were thus identified that did not have such deleterious effects. The *XcmI *restriction site, downstream of *REP2 *in the inverted repeat region (Figure 2), was one of several sites in the inverted repeat regions that did not appear to destabilize the plasmid under selective conditions (data not shown). We constructed pDB2711 by inserting a copy of *PDI1 *at this *XcmI *site with transcription in the same direction as *REP2*, in a plasmid otherwise the same as pDB2536 (Figure 2). DYB7 [pDB2711] gave a large increase in rTf expression in shake flasks (Figure 1, Lane C6). A similar effect was also seen in an alternative laboratory strain, JRY188, showing that this effect is not strain-specific (Figure 1, Lane C3). Fermentation titers of rTf produced from strains with single, double and multicopy *PDI1 *demonstrated that the increased productivity observed in SFC was also seen under production conditions, resulting in rTf titers of around 1.5 g/L (Table 1).

### Functional characterization demonstrates equivalence of rTf and plasma-derived Tf

**Figure 3:**
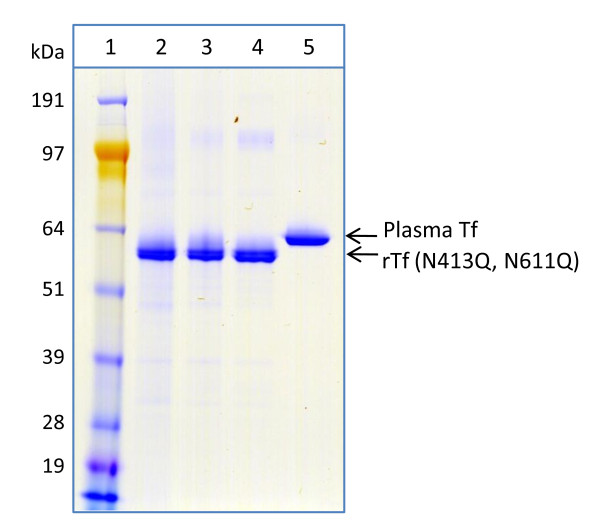
**Purification of rTf from high cell density fed-batch fermentation assessed by SDS-PAGE**. 1 μg samples were analyzed per lane by non-reducing SDS-PAGE (4-12% Bis-Tris NuPAGE^®^, MOPS buffer, Invitrogen). Track loadings were: Lane 1) SeeBlue^® ^Plus2 protein standards (Invitrogen) used for comparing relative migration of Tf samples only. Lane 2) DYB7 [pDB2711] fermenter supernatant. Lane 3) DYB7 [pDB2711] SP-FF eluate. Lane 4) DYB7 [pDB2711] DE-FF eluate. Lane 5) human plasma-derived transferrin.

rTf derived from the strain DYB7 [pDB2711] was purified using a two-step ion-exchange chromatography method for functional characterization. The purity of the protein was assessed by SDS-PAGE (Figure 3). Analysis of the product by urea-PAGE indicated correct iron binding with faster migration of the compact holo-form compared to the open structure of the apo-form (data not shown). The rTf product was also shown to be functionally equivalent to plasma-derived transferrin as a cell culture supplement [26]. Electron paramagnetic resonance (EPR) was also used to confirm correct co-ordination of the ferric ions in the rTf expressed from *S. cerevisiae *[27].

### Leader sequence optimization results in rTf product with the correct N-terminus

**Figure 4:**
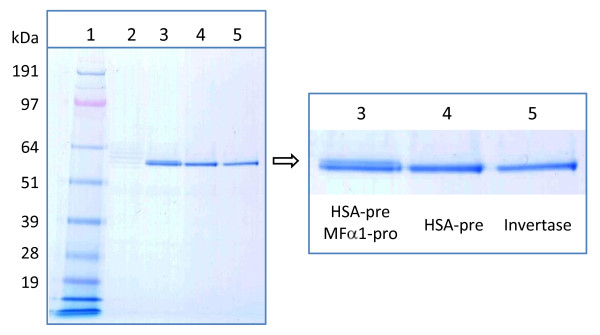
**Effects of alternative leaders on rTf N-terminus**. 3 μl of five-day BMMD SFC supernatant was analyzed per lane by non-reducing SDS-PAGE (4-12% Bis-Tris NuPAGE^®^, MOPS buffer, Invitrogen). Track loadings were: Lane 1) SeeBlue^® ^Plus2 protein standards (Invitrogen). Lane 2) DYB7 [pSAC35] negative control. Lane 3) DYB7 [pDB2711] with the modified HSA-pre and MFα1-pro leader. Lane 4) DYB7 [pDB2929] with the modified HSA-pre leader. Lane 5) DYB7 [pDB3557] with the *S. cerevisiae *invertase leader.

Despite the rTf product being functional, it was observed as a double band on SDS-PAGE gels (Figure 4, Lane 3), suggesting incomplete cleavage of the pre-pro leader sequence. The secretory leader employed in the construction of both pDB2536 and pDB2711 was a combination of a modified HSA-pre and *MFα1-*pro regions (with the sequence MKWVFIVSILFLFSSAYS↓RSLDKR↓) optimized for rHA secretion. N-terminal sequence analysis of the rTf protein secreted with this leader indicated that a proportion of the product carried the propeptide-derived hexapeptide extension (RSLDKR) - a result of incomplete cleavage by Kex2p.

**Figure 5:**
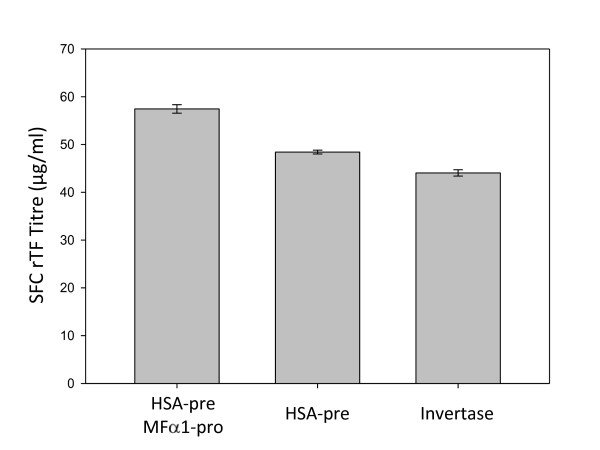
**Relative productivity of alternative leaders**. rTf (N413Q, N611Q) was expressed using three different leader sequences, mHSA/MFα1, mHSA and invertase, using 2 μm expression plasmids containing the *PDI1 *gene. Plasmids pDB2711, pDB2929 and pDB3557 were transformed into the yeast strain DYB7. The transformed yeast were inoculated at OD_600 _= 0.1 into BMMD SFC and grown for five days, after which supernatant concentrations were determined by HPLC. Error bars indicate standard deviations (n = 4).

An alternative leader sequence was constructed by deleting the coding region for the *MFα1*-derived pro-sequence hexapeptide and leaving only the HSA-derived pre-sequence terminating ...SSAYS (the "mHSA-leader"). The rTf product (from pDB2929) appeared as a single band when visualized by SDS-PAGE (Figure 4, Lane 4). However, N-terminal sequence indicated that there was still heterogeneity at the N-terminus, this time in the form of a YS dipeptide extension, suggesting unspecific cleavage by the yeast signal peptidase complexes. Consequently, the invertase leader, from the *S. cerevisiae *Suc2 protein, was used for rTf secretion as this has been used previously to produce recombinant proteins with the correct N-termini in yeast [28]. Again a single band was observed by SDS-PAGE (Figure 4, Lane 5), but in this case the N-terminus was found to be both correct and homogeneous. Analysis of SFC expression and protein sequence data for the three different leader sequences tested indicated that there was a trade-off between correct N-terminal cleavage and yield, with the invertase leader showing a 23% reduction in titer compared to the mHSA/*MFα1* leader (Figure 5).

For the commercial production of rTf the invertase leader sequence was used in combination with alternative N-linked glycosylation mutants (S415A, T613A as opposed to N413Q, N611Q) and codon optimization of the coding sequence. Fermentation of the resultant strain, DYB7 [pDB3237], under optimized conditions gave an average rTf titer of 2.29 g/L (Table 1).

### Strain selection and *PDI1 *overexpression increased rTf expression over fifty-fold in shake flask culture

**Figure 6:**
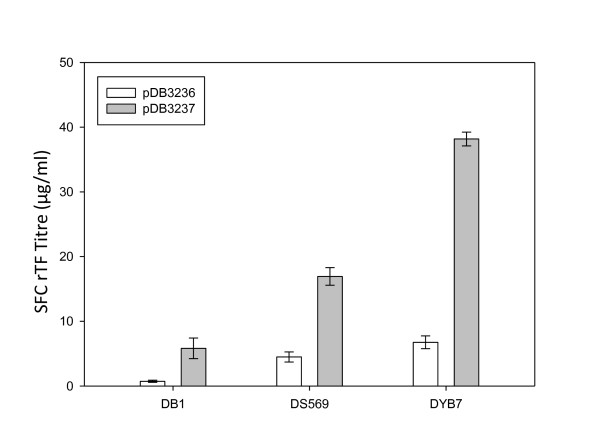
**Relative rTf (S415A, T613A) productivity of different *S. cerevisiae *strains**. The plasmids pDB3236 (-*PDI1*) and pDB3237 (+*PDI1*) were transformed into the strains DB1, DS569 and DYB7, which form part of the mutagenized strain series developed for rHA production. Yeast strains were inoculated at OD_600 _= 0.1 into BMMD SFC and grown for five days, after which supernatant concentrations were determined by HPLC. Error bars indicate standard deviations (n = 4).

In order to demonstrate the overall effect of strain selection and *PDI1 *overexpression, production of transferrin was analyzed in two progenitor strains to DYB7, with or without *PDI1 *on the expression plasmid (Figure 6). In SFC, DYB7, in the absence of *PDI1 *overexpression, showed more than a nine-fold increase in rTf production levels over the original progenitor strain DB1. When combined, *PDI1 *overexpression and strain selection resulted in more than a fifty-fold increase in rTf production. The increase observed with *PDI1 *overexpression in fermentation conditions was even greater than in SFC (approximately five-fold), with titers increasing more than twelve-fold (Table 1).

### MS profiling of rTf mutants confirms reduced glycosylation compared to plasma-derived transferrin

**Figure 7:**
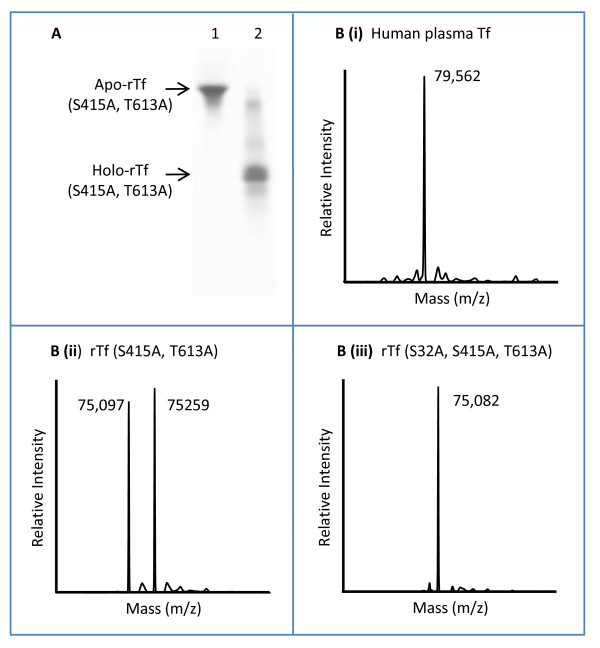
**Characterization of rTf. (A) **Urea gel analysis of rTf (S415A, T613A). 10 μg samples of purified recombinant apo-transferrin (Lane 1) and holo-transferrin (Lane 2) derived from DYB7 [pDB3237] were separated on 6% TBE Urea PAGE (Invitrogen) and stained with Coomassie G250 (Pierce). **(B) **Deconvolved mass spectra from analysis of plasma-derived and rTf samples using ESI-TOF mass spectrometry. i) Plasma-derived transferrin (Calbiochem), ii) rTf (S415A, T613A) derived from DYB7 [pDB3237], iii) rTf (S32A, S415A, T613A) derived from DYB7 [pDB3768].

The rTf product derived from the strain DYB7 [pDB3237] was characterized by urea-PAGE and mass spectrometry (Figure 7). As for the previous hexapeptide-extended form of rTf (N413Q, N611Q), urea-PAGE demonstrated functional iron binding. MS data showed the expected glycosylation differences between human plasma-derived transferrin and the rTf (S415A, T613A). The single predominant peak for the former (Figure 7B (i)) is likely to consist of a range of glycoforms. Heterogeneity in the recombinant product exists only in the form of O-linked glycosylation. The two peaks (Figure 7B (ii)) corresponded to the predicted mass of the non-N-linked glycosylated transferrin and a form containing a single hexose, probably resulting from yeast O-linked mannosylation at serine-32 [29]. Functional testing by receptor-mediated iron uptake (Table 2) and cell culture supplementation experiments [30] indicated equivalence between the plasma-derived and rTf analogue.

To control O-linked glycosylation, an additional transferrin mutant was produced to investigate whether serine-32 was the major site of O-linked glycosylation [29]. When analyzed by MS (Figure 7B (iii)), the rTf (S32A, S415A, T613A) produced a single peak at the predicted mass for non-glycosylated transferrin.

**Table 2:** Dissociation constants (K_d_) for transferrin receptor-mediated iron-55 uptake and unspecific uptake (B_max_) by K562 cells grown *in vitro *with recombinant and plasma-derived transferrins

Transferrin source	K_d _(nM)	B_max _(fmol/10^6 ^cells)
Human plasma Tf (Calbiochem)	105 ± 16	2668 ± 91

DYB7 [pDB3237]	104 ± 23	2543 ± 122

Errors indicate SEM (n = 3).

## Conclusions

The high expression level (> 2 g/L) achieved in high cell density fed-batch fermentation has enabled an economically viable process to be developed for production of a high quality transferrin analogue for cell culture purposes (Novozymes' CellPrime™ rTransferrin AF). The yeast-based fermentation process utilizes a chemically defined medium consisting of only salts, trace elements, sucrose, vitamins and ammonia. This fermentation process is much less costly than equivalent mammalian cell processes and, given the much higher yeast growth rate, is also more productive in relation to capacity utilization. In addition, the cGMP process avoids the use of animal-derived materials, thereby eliminating the risk of viral and prion contamination. Use of this rTf product in mammalian cell culture instead of animal-derived transferrin allows a simple elimination of these risks while continuing to deliver iron to cells via the natural mechanism of receptor-mediated endocytosis [26].

The system described here allows the production of transferrin or transferrin-related products for therapeutic use [1-5,7,9,11]. Proposed products include whole transferrin, adducts or conjugates (e.g. to diphtheria toxin for tumor therapy), and protein fusions. Other members of the protein family such as lactoferrin could be produced using similar approaches as well as derivatives such as mutant transferrins or truncated proteins. Transferrin fusions are of particular interest for two reasons: Firstly, the Tf/TfR interaction may provide an effective route for drug delivery to tumor cell targets; and, secondly, fusion to transferrin may allow the development of oral formulations for recombinant proteins. Thus we anticipate that the system described above will provide an enabling technology for a range of pharmaceutical products.

## Methods

### Plasmid and yeast strain construction

The human transferrin cDNA was excised from pcG13-Tf (kindly supplied by Robert W Evans) as a 2.37-kb *Pst*I DNA fragment and cloned into the unique *Pst*I site of pBST+ [20] to create pDB2449. The 5'-end of the transferrin cDNA was altered to replace the native transferrin secretory leader sequence with a modified version of the pre-pro HSA/*MFα1 *fusion leader sequence [31] consisting of the amino acid sequence MKWVFIVSILFLFSSAYSRSLDKR (referred to subsequently as the "mHSA/*MFα1*-leader"). Additionally, the 3'-end of the transferrin cDNA was modified to introduce two TAA translation stop codons. The transferrin sequence was then cloned between the *PRB1 *promoter and the *ADH1 *terminator in the plasmid pDB2923. The expression cassette from pDB2923 was subcloned as a *Not*I fragment into the yeast episomal expression vector pSAC35, in this case to produce pDB2506. To facilitate the expression of non-N-linked glycosylated transferrin, the asparagine residues at positions 413 and 611 were replaced with glutamine residues (CAA codon) by PCR mutagenesis to create the transferrin (N413Q, N611Q) expression cassette in pDB2536.

For genomic integration of *PDI1*, a *Pst*I-*Sca*I DNA fragment containing the *S. cerevisiae PDI1 *gene [32] was blunt ended and cloned into *Sma*I linearized pUC19 to create plasmid pAYE561. A 2.94-kb *Kpn*I DNA fragment from pAYE561 (containing the *PDI1 *gene) was inserted into YIplac211 at the *Kpn*I site to create pDB2389. Plasmid pDB2389 was linearized with *Bsu*36I for integration into the *PDI1 *locus of DYB7, creating the strain DP9.

In order to insert the *PDI1 *gene onto the expression plasmid, a linker containing a *Sma*I site was inserted into pSAC35 [23] at the *Xcm*I site downstream of the *REP2 *gene to create plasmid pDB2688. A 1.9-kb *S. cerevisiae PDI1 *fragment containing 212-bp upstream of the start codon and 148-bp downstream of the termination codon was then cloned, in the same orientation as *REP2*, into the *Sma*I site to create plasmid pDB2690. The expression cassette used to produce pDB2536 was cloned, using *Not*I sites, into pDB2690 to create pDB2711.

Leader sequence modifications resulting in the "mHSA-leader", lacking the final RSLDKR hexapeptide of the "mHSA/*MFα1*-leader" or the yeast invertase leader MLLQAFLFLLAGFAAKISA, were made to produce pDB2929 and pDB3557 respectively. Further sequence modifications to the transferrin sequence, including codon optimization of the open reading frame and reversed orientation of the *Not*I expression cassette, resulted in production of pDB3237. pDB3768 contains an S32A mutation in the transferrin coding sequence to control O-glycosylation. To investigate the effect of *PDI1 *overexpression, the *Not*I expression cassette from pDB3237 was cloned into pSAC35, resulting in production of pDB3235 (*Not*I cassette in same orientation as *LEU2*) and pDB3236 (*Not*I cassette in opposite orientation to *LEU2*). *Not*I cassette orientation had no effect on rTf production levels.

The yeast strains and expression plasmids used in this study are listed in Table 3 and Table 4 respectively.

**Table 3:** Yeast strains used in this work

Strain	Description/genotype	Reference
JRY188	cir^0 ^*MATa*, *leu2-3, leu2-112, ura3-52, trp1, his4, sir3, rme, GAL*	[36]

DB1	cir^0 ^*MAT*a, *leu2-3*, *leu2-112*	[19]

DS569	rHA-overproducing strain derived from DB1 by chemical mutagenesis. Known genotype: cir^0 ^*MAT*a, *leu2-3*, *leu2-112*	[19,20]

DYB7	rHA-overproducing strain derived from DS569 by chemical mutagenesis. Known genotype: cir^0 ^*MAT*a, *leu2-3*, *leu2-112 ubc4 ura3 yap3::URA3 lys2 hsp150::LYS2*	[37]

DP9	DYB7 with *PDI1*, *URA3 *and YIplac211 integrated at the *PDI1 *locus	This work


**Table 4:** Description of plasmids used in development of the rTf expression system

Plasmid	Description
pSAC35	2 μm-derived yeast episomal expression vector

pDB2506	pSAC35 with mHSA/*MFα1*-leader-rTf cassette at *Not*I site

pDB2536	pSAC35 with mHSA/*MFα1*-leader-rTf (N413Q, N611Q) cassette at *Not*I site

pDB2690	pSAC35 with *PDI1 *inserted at *Xcm*I site after *REP2*

pDB2711	pDB2690 with mHSA/*MFα1*-leader-rTf (N413Q, N611Q) cassette at *Not*I site

pDB2929	pDB2690 with mHSA-leader-rTf (N413Q, N611Q) at *Not*I site

pDB3557	pDB2690 with invertase-leader-rTf (N413Q, N611Q) at *Not*I site

pDB3237	pDB2690 with invertase-leader-rTf (S415A, T613A) at *Not*I site

pDB3768	pDB2690 with invertase-leader-rTf (S32A, S415A, T613A) at *Not*I site

pDB3235	pSAC35 with invertase-leader-rTf (S415A, T613A) at *Not*I site

pDB3236	pSAC35 with invertase-leader-rTf (S415A, T613A) at *Not*I site

### Culture conditions

Shake flask cultures with 10 ml BMMD (buffered minimal medium dextrose [20] in 50 ml conical flasks) were grown for five days at 30°C, 200 rpm. Shake flasks were inoculated to OD_600 _= 0.1 with two-day starter cultures of transformants grown in 10 ml BMMD in 50 ml conical flasks. Fed-batch fermentations were carried out in a 10 L Braun Biostat C fermenter at 30°C [33].

### Purification procedure

A two-step ion-exchange chromatography procedure was used to prepare rTf for protein characterization. An SP-Sepharose Fast Flow column was used as the first step, followed by a DEAE-Sepharose Fast Flow column as the second (GE Healthcare).

### Urea gel electrophoresis

Urea gel electrophoresis was performed using a modification of the procedure of Makey and Seal [34] with commercial minigels (6% homogeneous TBE Urea, Invitrogen). Apo-transferrin was prepared by dialysis against 0.1 M citrate, 0.1 M acetate, 10 mM EDTA pH 4.5. Reconstituted holo-transferrin was prepared from this solution by addition of 10 μl 1 mM FeNTA (prepared freshly as an equimolar solution of ferric chloride in disodium nitrilotriacetic acid) to a 50 μl aliquot and allowed to stand for 10 minutes to permit CO_2 _dissolution for completion of iron binding before electrophoretic analysis.

### ^55^Fe uptake competition in erythroleukemic K562 cells

For iron-55 uptake from labeled diferric transferrin, K562 erythroleukemic cells cultured in RPMI cell culture medium under standard conditions (bicarbonate-buffered, 5% CO_2_, antibiotics, 10% fetal calf serum) were washed with serum-free medium containing HEPES-buffer and 1 mg/ml of bovine serum albumin and used at a concentration of 10 million cells/ml in this medium. The samples tested were prepared as equimolar concentrations of apo-transferrin. Transferrin was loaded with iron according to a standard procedure using ferric nitrilotriacetate as the iron source [35]. Increasing concentrations of plasma-derived transferrin or the respective rTf sample (0, 25, 100, 200, 400, 800, 1600 nM), labeled with ^55^Fe, were mixed with 100 μl of medium. The reaction was started by the addition of 100 μl of cell suspension. A second series of parallel experiments was carried out in the presence of a hundred-fold excess of unlabeled diferric transferrin to account for unspecific binding. Since the counting data showed that iron uptake was linear during the first 30 minutes, the reaction was always stopped after 25 minutes at 37°C by immersion in an ice-bath. Three 60 μl aliquots of cell suspension were transferred to new tubes, and the cells were centrifuged in the cold and again after addition of an oil layer of diethylphthalate/dibutylphthalate. The supernatant was removed, and the cell pellet transferred into a counter vial and lysed with 0.5 M KOH + 1% Triton X-100. The lysates were neutralized with 1 M HCl after overnight lysis, mixed with Readysafe scintillation cocktail, and counted in the Packard Liquid Scintillation Counter. The results are presented as fmol ^55^Fe/million cells.

### Protein characterization

N-terminal amino acid sequence analysis was carried out by automated Edman degradation using an Applied Biosystems 494 Procise^® ^Protein Sequencer. Mass spectrometry samples were introduced into a hybrid quadrupole time-of-flight mass spectrometer (QqOaTOF, Applied Biosystems, QSTAR-XL^®^), equipped with an electrospray (IonSpray™) ion source in positive ion mode, using flow injection analysis (FIA), and, typically, 1 minute of spectral scans were averaged.

## Competing interests

Novozymes' authors declare competing interests as employees and/or inventors. RWE, DJT, HG, BSM and NT have no competing interests.

## Authors' contributions

CJAF initiated and coordinated the expression work and co-wrote the paper. TP completed the expression work, analyzed the data, and co-wrote the paper. JH produced the DP9 strain and performed expression work. ND, DJT and MJS performed MS, urea gel and N-terminal protein analysis. DW coordinated and analyzed fermentations. PM coordinated the purifications. RWE developed the project and characterized recombinant transferrin. HG, BSM and NT performed and analyzed iron-uptake assays. DS conceived the project and developed the yeast expression system. All authors have read and approved the final manuscript.
